# The Need for Governance by Experimentation: The Case of Biofuels

**DOI:** 10.1007/s11948-015-9729-y

**Published:** 2016-03-04

**Authors:** Lotte Asveld

**Affiliations:** Department of Values, Technology and Innovation, Delft University of Technology, Jaffalaan 5, 2628 BC Delft, The Netherlands

**Keywords:** Biofuels, Lock-in, Governance by experimentation, Learning effects, Values

## Abstract

The policies of the European Union concerning the development of biofuels can be termed a lock-in. Biofuels were initially hailed as a green, sustainability technology. However evidence to the contrary quickly emerged. The European Commission proposed to alter its policies to accommodate for these effects but met with fierce resistance from a considerable number of member states who have an economic interest in these first generation biofuels. In this paper I argue that such a lock-in might have been avoided if an experimental approach to governance had been adopted. Existing approaches such as anticipation and niche management either do not reduce uncertainty sufficiently or fail to explicitly address conflicts between values motivating political and economic support for new technologies. In this paper, I suggest to apply an experimental framework to the development of sustainable biobased technologies. Such an approach builds on insights from adaptive management and transition management in that it has the stimulation of learning effects at its core. I argue that these learning effects should occur on the actual impacts of new technologies, on the institutionalisation of new technologies and most specifically on the norms and values that underly policies supporting new technologies. This approach can be relevant for other emerging technologies.

## Introduction

The use of biomass for the replacement of fossil resources has been hailed as a sustainable solution that might help combat both climate change and pollution (OECD 2009; EC 2012). The bio-economy is an economy where biomass is the main resource for energy, materials and chemicals and this resource is used with the utmost efficiency. Biomass can be obtained from food and feed crops, non-food crops and various types of wastes and residues.

However one of the first and most prominent applications of biomass: first generation biofuel, is surrounded with controversy. The sustainability claims attached to biofuels by policymakers and producers (EC 2006; EBB 2014) have been questioned by environmentalists and scientists (such as EEA 2006; Gallagher Review 2008; Searchinger 2009; Croezen et al. 2010; Plevin et al. 2010). In the face of this criticism, some of it well documented, the European Commission has tried to alter the course of its policies but met with fierce resistance from some member states, and fierce support from others. The resistance stems from the economic interests of parties that have invested in (unsustainable) biofuels and countries that see economic opportunities in (unsustainable) biofuels.

Eventually the European Commission arrived at a policy proposal that reduces support for biofuels, but considering the evidence on the sustainability effects of first generation biofuels, this support might have been reduced even more, as was initially proposed by the European Commission. This political failure to assure the sustainability goals that originally motivated the biofuels policies, gives cause for reflection on the governance of other uses of biomass. How should policies be designed to assure that the use of biomass is indeed sustainable?

The social resistance towards adaptation of existing policies in support of biofuels, indicates a classical Collingridge dilemma. This dilemma states that when a technology can still be easily changed, at the beginning of its use, so little is known about its possible consequences that social actors can’t adequately anticipate that. Therefore they cannot avert any unwanted consequences at this stage by adapting the technology. Once a technology is widely used, the consequences become known, but at that stage it is very difficult to adapt the technology to avert unwanted effects because the technology has become socially entrenched (Collingridge 1980).

To avoid such lock-ins, Collingridge himself saw two solutions: one is to increase knowledge at earlier stages of the development of a new technology and the other is to increase social control over technological trajectories (Nordmann 2010). However, as we will show, existing approaches along these lines do not suffice in the context of the bio-economy.

Existing approaches such as anticipation, adaptive management and strategic niche management either do not reduce uncertainty sufficiently or fail to explicitly address conflicts between values motivating political and economic support for new technologies, as will be discussed in “Strategies to Prevent a Lock-In” section. In this paper, I suggest to apply an experimental framework to the development of sustainable biobased technologies. This framework has been proposed by Van de Poel (forthcoming). Such an approach can serve to increase the social control over a specific technology, even if it is surrounded with a considerable degree of uncertainty. As such it has relevance beyond the scope of biofuels, but might also be applicable to other emerging technologies.

These uncertainties can most likely not be completely reduced, but strategies can be deployed to deal with them and to accommodate learning effects in supporting governance structures. These governance structures should thus allow for adaptation to new insights. What is specifically needed is more attention for the explication of norms and values motivating policies in support of new technologies and learning effects thereon.

I will first discuss the case of biofuels and the uncertainties present there. Then I will discuss how the existing approaches of anticipation, adaptive niche management and strategic niche management might have been applied to this case and I will delineate the addition of the experimental approach. At the end of this paper, I will arrive at some guidelines on what is required for such an experimental approach that can be useful for the development of other technological applications.

## The Biofuels Experiment

### Biofuels Policies

Biofuels are fuels for transport produced from biomass. The majority of presently used biofuels are the so-called first generation biofuels, made by fermentation of sugars stemming from edible forms of biomass, such as corn, sugarcane or rapeseed. More advanced forms of biofuels, also referred to as second and third generation, use different feedstock for the production process, such as the agricultural waste (straw, stems, leafs, etcetera) of food and feed crops or dedicated ‘energy’ crops (such as Eucalyptus or Jatropha) or are produced with different means such as through algae and cyanobacteria.

Biofuels were first hailed as a green solution for climate change as well as an opportunity for sustainable development, economic growth and energy self-sufficiency (DOE 2010; EC 2006). Both the United States and the European Union have developed policies supporting these biofuels. In 2003 the European Commission issued a directive that made it mandatory to blend in biofuels with traditional fossil fuels.

In 2010 this directive was replaced with another directive that obliged member states to derive 10 % of their energy for transport from renewable resources in 2020. Most member states use first generation biofuels for this because they are widely available. Aside from these directives the EU also supports biofuels through the use of subsidies.

From the beginning of their use, biofuels have been surrounded by uncertainties, about their physical impact, as well as their moral evaluation and their institutional embedding (Asveld et al. 2011). These uncertainties have been wide-ranging and difficult to deal with for policymakers, eventually leading to a policy lock-in. The policies instigated the developments of biofuels. Without the policies the biofuels would have never developed to such an extent as they did. Biofuels have no advantage over other fuels aside from the advantages ascribed to them by policy-makers.

### Three Levels of Uncertainty

Uncertainties about the impact of new technologies usually exists at three distinct but related levels: physical, institutional and moral (cf vd Poel forthcoming). The physical impact refers to the measurable risks and benefits of a specific technology, such as its environmental impact. The institutional impact refers to the kind of social structures needed to adequately embed a technology. We consider institutions to be the formalisation of a consensus between multiple actors (cf Bachmann and Inkpen 2011), with sufficient legal or moral status to impact on the further development and use of relevant technologies. Examples are the regulation of risky technologies, subsidy schemes or voluntary schemes for the monitoring of sustainability effects. Moral impacts refers to the norms and values we use to evaluate a technology. In some case the technology may have such an impact that the prevalent norms shift. Social media have for instance shifted our perception on the value of privacy. Many people appear to value other things, such as being in touch with friends continuously, over privacy in the context of using social media.

#### Physical Uncertainty

When biofuels policies were developed and reinforced in 2010, they were based mainly on the assumption that first generation biofuels would reduce the emission of greenhouse gasses, even though evidence suggesting the opposite effect was already available (Gallagher 2008). Whether they would actually reduce the emission of greenhouse gasses, was still uncertain.

Since then, the wide-spread use of biofuels has revealed much of the actual sustainability effects of these technologies, such as the CO_2_ emission associated with divergent forms of biofuels, and the infamous practice of land grabbing, referring to Western companies obtaining land for the production of biomass for biofuels that was previously used for the production of food by local inhabitants (Kelly 2012; Cotula et al. 2009).

In line with the Collingridge dilemma these physical effects became known, to some extent at least, only once the biofuels were widely used and their effects became measurable in a meaningful, large-scale manner. These effects continue to be disputed, but it is a dispute supported with evidence, beside claims based on ideology (see amongs others Kelly 2012; Hamelinck 2013; Helaine et al. 2013). This evidence on these various issues even caused the UN climate panel (IPCC) to change its views on biofuels from positive to cautious in its latest report on climate change (IPCC 2014).

One of the crucial, contested issues in establishing the actual sustainability effects of first generation biofuels, has been the inclusion of Indirect Land Use Change (ILUC). Some actors in the debate argue that the use of biomass for any application besides food will lead to the clearance of land elsewhere to make up for the loss of the land that is now used for other applications. This holds especially true for first generation biofuels, which are sourced from the edible parts of plants. This indirect land-use change produces a high amount of CO_2_, so much so that it may make biofuels less climate friendly than fossil fuels. CO_2_ is released when trees that store carbon are cut and burnt.

According to some other parties this ILUC-effect does not take place or at least not in at any meaningful scale. They claim that the clearing of land is influenced by so many different factors such as developments in the world food market, that a direct relationship with biofuels cannot be established (see for instance Biodiesel Magazine 2014). However more and more evidence is available to suggest that this effect does indeed take place (Searchinger et al. 2008; Croezen et al. 2010).

#### Institutional Uncertainty

These continuing new insights on the sustainability effects of biofuels, have led the European Union to reconsider its policies numerous times. For instance the original obligation to blend-in biofuels issued in 2003, was replaced in 2010 with the obligation to derive 10 % of the energy used for transport from renewable sources, thereby allowing other sources of energy such as hydrogen or electricity to compete with biofuels. Both of these directives were heavily criticised for not taking full account of the adverse sustainability effects of biofuels (Koppejan and Asveld 2011), indicating that these institutions remain contested.

Other forms of institutionalisation also continue to invoke debate. Besides changes in legal obligations, an important institutional approach has been to develop sustainability criteria. For its 2010 directive on renewable energy for transport the European Commission issued such criteria: only those resources that lived up to these criteria could be considered as fulfilling the obligation. The most important criterium is that CO_2_-emissions should be reduced by 35 % as compared to traditional fossil fuels. This percentage goes up to 50 % in 2017 and 60 % in 2018.

However many organisations have criticised these criteria for being too lax. The disagreement is fuelled by the many uncertainties that still surround the physical impact of biofuels. Due to these uncertainties and the varying perspectives on what issues are the most important, many organisations have developed their own sets of criteria, which are mostly voluntary. The G8 has for instance issued its own guidelines, as well as many European countries. In the Netherlands the NTA8080 can be used by companies that want to support their sustainability claims. These criteria often do not take into account ILUC, but they may explicitly exclude the use of edible biomass and often include aspects such as biodiversity and social and economic impacts that are not part of the EU criteria (NEN 2014; Schlamann et al. 2013).

#### Moral Uncertainty

In relation to the moral impact of biofuels, there is uncertainty about which values and which norms should be used to evaluate this technology. Biofuels have met with fierce resistance and stern criticism from the outset for instance because the first generation biofuels are made from the edible parts of plants such as the kernels of corn. This has led to public outcry with the slogan ‘food for fuel’, referring to the paradox of food for fuel as a sustainable solution while plenty of people in this world are hungry. The moral dictate that food should always be used to combat hunger clashed with the (less prominently voiced) value that farmers all over the world, and specifically in Europe and the United States should have an extra source of income to assure the continuing existence of a productive farming industry (Koppejan and Asveld 2011). This latter value has not been articulated as prominently within the European Union as has the value of sustainability, but the political choices that have been made suggest that it did play a central part in the design of the relevant policies (Sharman and Holmes 2010).

Additionally there is continuing uncertainty about the interpretation of sustainability and therefore about sustainability norms. In the debate on the bio-economy at large, often various perspectives emerge on what sustainability amounts to. These perspectives can be linked to worldviews in which perceptions on nature and our relation to nature play a central part along with other related issues such as how we should organize the economic system and how decisions should be taken (Asveld et al. 2014). Such worldviews give rise to often intractable disagreements that can be recognized in the specific debate on biofuels. To recount some of the issues at stake in the discussion on the sustainability of biomass: Can large scale plantations or large scale production plants be considered sustainable? Can the control of big multinational organisations over biomass resources be considered sustainable? Can the use of advanced genetic technologies be considered sustainable and if so, under what conditions? These issues continue to be a source of debate.

### Policy Lock-In

The failure to accommodate all these uncertainties eventually led to a policy lock-in. When confronted with mounting evidence of the detrimental effects of the use of biofuels (Euractiv 2012a), some of it even coming from its own scientific institutions (Euractiv 2012b), the European Commission proposed in 2014 to put a cap of 5 % on the use of first generation biofuels for its renewable energy target (Euractiv 2012c). However this proposal met with stern resistance from some of the member states and the biofuels industries forcing the Commission to raise its cap to 7 % and to disregard any ILUC-effects in the accounting of CO_2_-emissions. This has since then remained the leading proposal (Euractiv 2015). The proposal is due to be discussed in the European Parliament in 2015.

The 7 % cap and the disregard of ILUC in the current proposal has been greeted as victory by the biofuels industry, because it gives a clear guideline for the industry and because it allows it to expand its production beyond their current levels. Its argument is that ILUC does not occur when the biomass is produced within Europe itself (Euractiv 2013a). Environmental organisations are less happy, but concede that a 7 % cap is better than nothing.

The above example shows how a policy for sustainability can create its own lock-in. The learning effects on the sustainability impact of first generation biofuels could only partially be accommodated in the policies due to political opposition from actors with vested interests. As a representative from the biofuels industry stated: more stringent reformulation of the biofuels policies would be unfair to those who have already invested in biofuels (Euractiv 2012a). Support for accounting for ILUC-effects in the CO_2_-footprint of biofuels came from only four European member states, with many others opposing it (Euractiv 2013b). The interests of the member states opposing ILUC have been identified by many as coinciding with the ‘sugar industry’, implying that they harbour many sugar producing farmers (such as sugarbeets) that need a steady market for sugar such as biofuels (Sharman and Holmes 2010).

## Strategies to Prevent a Lock-In

How could this opposition from vested interests have been avoided? A lock-in such as described above suggests that the control over the further development of a technological trajectory has become problematic. The political institutions supposed to steer a technology towards its presupposed goal, namely sustainability, fail to do so. This failure results from the various kinds of uncertainties that confronted the policy-makers involved. They couldn’t properly accommodate the sustainability effects because the evidence for them remained inconclusive. They weren’t clear on the dominant values that they should use to assess this technology and as a result they failed to design institutions that could. This uncertainty kept politicians and policymakers from adapting the policies to safeguard the predefined policy targets. This in turn allowed existing industries such as the so-called sugar industry to establish a firm grip on the production of biofuels technology and the framing of its benefits.

As mentioned above, Collingridge envisioned two possible solutions to the dilemma he formulated: increasing knowledge at early stage of a technology and enhancing the social control over technological developments. We will now discuss how useful these approaches might have been in preventing the biofuels lock-in.

### Anticipation

Increasing knowledge at early stages of the development of a technology would boil down to anticipation. Anticipation is an integral element of various approaches to dealing with new technologies, such as (Constructive) Technology Assessment (Schot and Rip 1997), risk assessment (Haimes 2009) and most recently Responsible Research and Innovation (Stilgoe et al. 2013). Well informed anticipation that includes a variety of perspectives may potentially substantially reduce uncertainty and thereby enable policy makers and designers of technology to avoid unwanted consequences.

However anticipation is always necessarily limited. Unexpected consequences may emerge even after the most thorough and inclusive anticipation efforts. As van de Poel (forthcoming) shows, this is due to the complex epistemological uncertainties that surround a new technology, i.e. technologies can have impacts at so many different levels that it is often impossible to predict all of them correctly, also because many of these effects depend on how individuals will eventually apply a new technology and what (moral) meanings they associate with a technology.

Collingridge himself did not expect much from the strategy of anticipation because it requires unbiased experts. He thought such experts do not exist because experts are always also participants in society with their own interests and values (Nordmann 2010).

In a more elaborated argument in the same vein, Sarewitz (2004) claims that scientific evidence lies at the heart of many value disputes that underpin many political debates. Scientific evidence is always inevitably inconclusive, Sarewitz states, because the evidence that is provided will come from various scientific disciplines and sub disciplines that each provide varying framings of the issue at hand. These in turn result from varying assumptions about causal relationships and what issues deserve most attention. This is not to say that there can be no scientific agreement on any issue at all. On the relation between climate change and carbon emissions for instance, there is a considerable scientific consensus, even though the evidence remains inconclusive.

What should be noted however is that the inconclusiveness of scientific evidence allows politicians to pick whatever scientific evidence is most congruent with their specific political position. Moreover, scientific evidence does not indicate what political choice should be made, that remains the responsibility of the politician. The dynamic of this can be recognized in the political developments around biofuels.

Prominent adverse effects had been anticipated for biofuels, but this did not prompt political action. There have for instance always been suspicions towards the existence of the ILUC effect (Gallagher 2008; Croezen et al. 2010; Plevin et al. 2010), voiced mainly by environmental organisations (FoE 2008) but there wasn’t sufficient evidence to back it up, as Christine Hedegaard, former Commissioner for Environmental Affairs declared:

Responding to a question from EurActiv, Hedegaard said that the EU had acted judiciously in 2009 by incentivising biofuels, while leaving the door open for future measures on ILUC.“Everyone was aware that there might be such a thing as ILUC, but the science at that time was not very well developed,” she said. “It was not a mistake that it wasn’t done at that time.” But “the time is here” now, she added. (Euractiv 2012c)

In the above quote Hedegaard refers to her proposal to put a cap on biofuels from edible sources of 5 %, which has since been watered down to 7 % and is due to be discussed in the European Parliament.

When the biofuels policies were designed in 2003 and followed up on in 2010 it was anticipated that biofuels from edible sources would reduce greenhouse gas emissions, although there was no conclusive evidence supporting that assumption, moreover there was even evidence to the contrary, but that did not hinder the manifestation of political action. This shows how anticipatory evidence can both support or distort political action, depending on the political preferences at the time.

Another example shows how recommendations from scientists to avert adverse consequences can be interpreted according to political whim. Many of the reports that were critical of the use of biomass called for a better methodology to calculate emissions of biofuels use and advised that we focus on biomass that looks most promising in terms of sustainability, namely waste biomass, such as waste edible oils, cellulosic biomass and biofuels from alternative sources such as algae (such as EEA 2006; Croezen and Kampman 2008). This has been translated by politicians into a mandate to stimulate edible biofuels in the expectation that this would eventually provide a stepping stone for more ‘advanced’ or ‘third-generation’ biofuels (Euractiv 2013b) and would lead to learning effects with regard to a better methodology to calculate the emissions from biofuels (Koppejan and Asveld 2011). Instead they might have opted to abandon political support for first generation biofuels, but obviously chose not to.

This ‘cherry-picking’ by politicians from the available scientific evidence is not limited to the stage of anticipation, because evidence is rarely conclusive beyond doubt even when it concerns large scale phenomena (Sarewitz 2004; Slob and Staman 2012). Much of the evidence about the CO_2_-emissions of biofuels remains inconclusive even when it’s use has been widely spread. More evidence points to the existence of ILUC effects, but reports with contrary evidence also keep emerging, with different actors with different interests highlighting different aspects of the emerging knowledge.

But the available evidence does become more solid and convincing, which makes cherry-picking more difficult. Additionally, insights into institutional embedding and moral evaluation also progresses which reduces uncertainty in those areas. This will have an effect on how liberally the scientific evidence can be interpreted by politicians. We will return to this point below.

### Resilience and Regime Change

An approach that seeks to both enhance social control while reducing uncertainty is that of resilience through diversity. For the effective management of natural resources as well as the realisation of sustainability related policy goals, authors in the fields of evolutionary economics, evolutionary policies and adaptive management have proposed the condition of resilience through diversity (Rammel and van de Bergh 2003; Nill and Kemp 2009). Such resilience implies that in any given case, a wide range of solutions is applied to avoid a lock-in, i.e. the system at hand is made adaptable through diversity. If one solution does not work out, another is readily available.

The various solutions are executed on a small scale only. This resilience enhances the ability to adapt to changing circumstances while it provides at the same time insights into the consequences of these varying solutions. The ability to adapt would have to be supported by learning effects of the various actors involved so that the system is not only technologically flexible but also socially adaptable. Ideally a wide range of actors is invited to co-design management and policy practices as this strengthens the knowledge basis for consequent adaptions, i.e. it accommodates learning effects and generates wide social support (Berkes 2009).

Such an approach seems applicable to biofuels since they can be sourced from a variety of feed stocks. First generation biofuels are derived, among others, from corn, sugar cane, sugar beet, hemp, soy, sunflower, rape seed, waste oil and palm oil. Some of these feed stocks perform better in terms of sustainability than others: sugar cane and waste oil stand out in terms of net CO_2_ gain if ILUC is included compared to the others feed stocks (Croezen et al. 2010; Plevin et al. 2010). Applying a wide variety of varying feedstocks allows the specifics about each of these feedstocks to be brought to the fore.

Additionally the policy formulated by the EU in 2010 allows for a wide range of technologies to be employed to reach the target of 10 % energy from renewable sources in transport in 2020. Member states can also stimulate the use of electrical cars or hydrogen cars to meet their target.

However, if such a variety of technological solutions are explored on a small scale only, as is often proposed, this will not reduce uncertainty at all three levels identified earlier. If biofuels had been produced in small amounts only, this wouldn’t have involved the wide range of actors it involves now and therefore it wouldn’t have brought out all the clashing perspectives that fuel the current debate. It is also questionable whether the ILUC-effect, which is crucial in establishing the limited effect on CO_2_-emissions, could have been determined with any accuracy if biofuels were produced on a small scale only. Additionally such small scale applications probably do not require much institutional embedding and therefore any reduction in uncertainty to this effect would not have occurred.

To address such broader issues along with stimulating diversity, approaches such as transition management (Loorbach 2007) and the associated Multi Level Perspective (MLP) (Rip and Kemp 1998; Rotmans et al. 2001; Geels 2005) have been developed. These approaches build on the stimulation of a variety of small scale (sustainable) technologies, which are termed ‘niches’. Other relevant levels consist of the socio-technical landscape and the socio-technical regime. Both the regime and the landscape refer to the social embedding of technologies, where the regime refers to more tangible instruments such as pricing, taxes, regulations and standards and the landscape refers to broader political and socio-cultural attitudes and trends (Geels 2004). As such the regime might be considered to overlap with the institutional level I mentioned earlier and the landscape with the level of morality.

The Multi Level Perspective (MLP) originated as a descriptive tool for the analysis of historical transitions. It has been used since then in the creation of management models for societal change such as Strategic Niche Management (SNM) that seeks to actively stimulate societal change (Schot and Geels 2008). The niches are considered as ‘seeds’ for change at the other two levels. The ‘seeds’ challenge the incumbent powers. By organising protected spaces for the niches, new sustainable technology gains social power and can challenge the social technological frameworks in the existing regime. Such an approach might have proven useful for the production of biofuels. In the case of the biofuels the European Union might have gained greater adaptability if it would have allowed for diversity initially only at a small scale, at the first stages of developing a biofuels industry.

However what remains unexplained in this approach is how the existing institutions might have allowed for the approach of strategic niche management in the first place. In the words of the Multi Level Perspective: how does it become possible to influence the actors in the current regime to open up to seeds of change that might threaten the existing socio-economic order?

Actors presiding in incumbent regimes may exercise their power to prevent any niche experiments to ever blossom to their full potential (Smith et al. 2005). Existing institutions and socio-economic configurations (or regime and landscape) considerably influenced, and continue to influence how biofuels are to be applied, thereby hindering the pathway to a sustainably optimal use of biofuels. An obligation to blend in biofuels existed at the European level from 2003 onwards, while there was still little knowledge on sustainability effects. This was probably due to lobbying from agricultural actors (Sharman and Holmes 2010). After that, market dynamics dictated which feed stocks were attractive. Producers and national governments opt for those feed stocks and technologies that are readily available and easy to blend into existing infrastructures such as corn, rape seed and sugar beet within Europe. Investments were made to produce biofuels from these feed stocks at least as much as from the more sustainable sources, such as sugar cane. These vested interests promoting the less sustainable feed stocks eventually prevented a rigorous adaptation of existing biofuel policies.

In relation to this case, it appears that the strategic niche management (SNM) and related transition management approach do not entirely accomodate some of the competing values and economic interests that may also influence the development of the niches and their subsequent impact on regimes and landscapes. SNM focusses on change from an unsustainable to a sustainable society, thereby taking the value of sustainability as the main motivating value for the niche experiments, assuming it’s motivating position to be beyond the need for public scrutiny (cf Shove and Walker 2007). The existence of various perspectives and visions on what sustainability amounts to is recognized, but moral learning with regard to these perspectives or its balancing with other values is not given a explicit part in the transition. SNM also assumes that the niches will produce mainly desirable or sustainable results, thereby overlooking competing societal dynamics that also influence the niches and their outcomes (ibid). In the experimental approach, competing values and interests are a main object of learning to increase social control over technological developments.

## An Experimental Approach

To overcome the pitfalls mentioned above, an experimental approach has been proposed by Van de Poel (this volume) that builds on the insights gained from the approach of strategic niche management and that of resilience through diversity. According to Van Der Poel we have an obligation to consider the introduction of new technologies as an experiment, because this acknowlegdes the many uncertainties present when a new technology is introduced into society. An experiment is always conducted with the explicit aim of learning. The aforementioned approaches offer tools for learning about impact and institutional effects. The experimental approach adds the notion of moral learning, i.e. learning about how values play out in practice, what relevant different interpretations of values abound and how values, or the balance between values might change due to the introduction of new technologies.

Furthermore the notion of experiment suggests that it is possible to cancel, i.e. that the distributed applications of a technology is stopped or at least adjusted, or that people can withdraw if the results are socially unacceptable (Jacobs et al. 2010).

### Moral Learning

To enable such an approach requires not solely learning processes about the impact and institutionalisation of new technologies, as is offered by adaptive management, transition management and strategic niche management, but also about values motivating support for technological developments, understandings of those values and consequently the norms by which we evaluate technologies. Explicating values and drawing lessons on how to implement those values are important tools in determining the social acceptability of new technologies. If these values and goals are clear, they can provide a clear measuring stick against which the development of new technologies can be assessed.

In the case of biofuels, competing values motivated the supporting policies: one was sustainability and the other increased income for European farmers. Of these values, sustainability was clearly articulated while the other: increased income for farmers, was less clear.

Up to a certain point, the co-existence of two motivating values was an asset to the development of biofuels, because in this way resources (financial and material) were mobilized. The farming community was willing to co-operate and to invest because their interests were taken to heart. Such support is needed for any technology to move beyond a small scale application.

However these values turned out to be incompatible in the long run. The clash over ILUC is basically a clash between these two values. The agricultural lobby seeks to protect its investment and economic prospects, the environmental lobby seeks to guarantee a positive sustainability impact of the use of biofuels. A lock-in might have been avoided if the possible contradictions between these lobbies and their values had been explicated and addressed from the early stages of the development of biofuels (cf Laak et al. 2007). It would have enabled policy makers to develop institutions to accommodate both values.

Explicating both values would have also increased public control over biofuels, and this would in turn enhance political control. Since not only financial support but also legitimacy is required to stimulate a technological trajectory (Smith et al. 2005), the values that explicitly motivate technology related policies require public support. The biofuels policies were legitimized through the value of sustainability. Although the development of the biofuels got a head start because of the support of the agricultural lobby, this support now risks to undermine the legitimacy of the policies as it undermines the claim of the sustainability of the biofuels. Had the underlying values been made explicit, they would have come under public scrutiny which could have led to robust policies with wide-spread social support.

### Learning About Impacts

Knowledge offers another avenue for social control. In an experimental approach, this can be done first of all by copying the approach of resilience through diversity. Various variations on the methods of production and sources of biofuels can be explored on a small scale to get an initial idea of their effects without risking lock-in effects. Thereafter, promising biofuels can be produced in ever larger quantities. Following the approach of adaptive management, learning may take place at consecutive scales of application, such that each scale builds on the previous scale by enlarging the scope in both moral, physical and institutional aspects.

The learning effects that emerge from this investigation can be accounted for in sustainability criteria. These criteria themselves are potentially highly flexible: they can be adapted to and integrate any new insights resulting from the use of biomass. New criteria can be developed when learning gives rise to the necessity for new criteria.

Since these criteria are usually developed with the inclusion of a variety of actors the risk of dominance by one specific interest shaping knowledge in a specific direction is mitigated and epistemic quality is enhanced. This approach also follows the adaptive management approach. The actors involved often include environmental NGO’s, financing institutions, energy companies and farmers, as is done in the Dutch platform NEN and the Committee Corbey. However, actors from the global South are not always represented so learning can be expected to improve when these actors are also present (Partzsch 2011).

### Institutional Learning

A way to prevent the development of technological trajectories that are closely aligned with a specific interest or value is to organise policies around a specific goal instead of a technology. Technology itself can be used to achieve social control. As is also recognized in the approach of strategic niche management, technology can be the outcome of social processes, but it also reinforces the motivations and interests behind these social processes thereby excluding alternative technologies.

Policies around biofuels had biofuels as their main target. Alternative policies might have been the reduction of CO_2_-emissions for transport. The European Commission changed its strategy once the biofuel industry came up to speed, to an obligation for member states to derive 10 % of their transport energy from renewable sources, which is a much more generic target than obliging member states to blend in biofuels with traditional fuels. However, since this policy was issued only after biofuels had become widely available, most actors choose to use biofuels to meet this obligation, foregoing alternatives such as electric transport or hydrogen cars.

Formulating a specific target allows manufacturers to choose their own course. This may stimulate a wide variety of approaches, in line with the dictates of the resilience and niche management approach. These can be supported if they meet the target of reducing CO_2_.

Whether this target is indeed reached, can only be established once the technology is developed on a substantial scale. The question is whether the risks of investing in a technology that might not turn out to be feasible should be shouldered solely by the investors. Public institutions such as universities should receive sufficient support to investigate the effectiveness of various technological trajectories. This would not imply support for a specific technology, but it does imply support for the process of learning.

## Conclusions

Policies stimulating the use of biofuels have led to a lock-in. Confronted with evidence that first generation biofuels were not sustainable, the European Committee proposed to put a cap on the use of these biofuels but had to water it down after resistance from a substantial number of the member states.

Such a lock-in might be avoided for other technologies, such as other biobased applications by applying an experimental approach to governance. This approach seeks to reduce uncertainties about the possible adverse effects of emerging technologies and to enhance social control over technology to make adaptability possible. This approach builds on existing efforts to avoid a lock-in such as strategic niche management and adaptive management. It copies lessons from these approaches about institutional learning and learning about impacts, but stresses the need for moral learning.

This kind of governance is intended to prevent a lock-into an undesirable technological trajectory. It is based on two strategies: reducing uncertainty about the impacts of a technology and enhancing social control over technology to enable the adaptability of a technological trajectory.

A few guidelines can be formulated for governance by experimentation for the use of biomass. Governance by experimentation should organise small scale exploration of new technologies and be built on the knowledge that is derived from this small scale application. The applications that appear promising can be scaled up, both in quantity and in institutional support, i.e. in the form of tax exemptions and subsidies.

Knowledge about the effects of new technologies should be produced in collaboration with a wide range of actors. This approach has proven useful in the context of biofuels and with other technologies that impact on the natural environment, i.e. with approaches of adaptive management. This approach avoids the dominance of one particular perspective in the shaping of knowledge and thereby increases social control over that technology.

The values that motivate policies and other institutional supports for biobased technologies should be clearly explicated so that they are open for public scrutiny. Additionally, policy goals should be formulated in generic terms instead of focussing on a specific technology.
